# Gamma/Delta T Cells and Their Role in Protection Against Malaria

**DOI:** 10.3389/fimmu.2018.02973

**Published:** 2018-12-20

**Authors:** Katrien Deroost, Jean Langhorne

**Affiliations:** Francis Crick Institute, London, United Kingdom

**Keywords:** gamma/delta T cells, malaria, activation, human, mice, skin, liver, red blood cells

## Abstract

Whether and how γδT cells play a protective role in immunity against *Plasmodium* infection remain open questions. γδT cells expand in patients and mice infected with *Plasmodium* spp, and cytokine production and cytotoxic responses against blood-stage parasites are observed *in vitro*. Their expansion is associated with protective immunity induced by irradiated sporozoite immunization, and depletion of γδT cells in some mouse models of malaria excacerbates blood-stage infections. It is now clear that these cells can have many different functions, and data are emerging suggesting that in addition to having direct parasitocidal effects, they can regulate other immune cells during *Plasmodium* infections. Here we review some of the historic and more recent data on γδT cells, and in light of the new information on their potential protective roles we suggest that it is a good time to re-evaluate their activation requirements, specificity and function during malaria.

## Introduction

Malaria is endemic in large parts of tropical and subtropical countries with high morbidity and mortality. After a period of decline in the number of cases each year, malaria incidence is on the rise again, partly because of increased resistance against drugs and insecticides. Effective vaccines for malaria are therefore urgently needed. Whole organism vaccines, such as those containing irradiated sporozoites are promising candidates, and could confer sterilizing immunity against *Plasmodium falciparum* (1, 2). The mechanisms of protective pre-erythrocytic immunity are not fully elucidated, but are commonly assumed to be mediated by antibodies and CD8^+^ T cells (3). Recently, however, a subset of T cells carrying γδT cell receptors (TCRs) has been shown to associate with protection induced by irradiated sporozoites (4). This observation has sparked a renewed interest in the potential role of γδT cells in protective immunity and immunoregulation in malaria. Studies of γδT cells in malaria were first published nearly 30 years ago, and since then there has been substantial progress in understanding the biology of these cells. However, relatively little research has been done applying this more recent knowledge to the investigation of malaria immunity. Here we review some of the historical literature on γδT cells in malaria in both human studies and experimental models of malaria in the context of more recent findings on development, function and recognition of these cells in the hope that it spurs more widespread interest in their possible role in malaria.

## γδT Cells

Until recently, it was thought that γδT cells were simply innate immune T cells with limited or somewhat redundant functions. The current view is that these cells complement many different players of the immune defense system (5), and, it is becoming clear that they are heterogeneous populations of cells with important unique roles in many infections, autoimmune diseases, allergies and in immunoregulation. To understand what they do in malaria, it is important to understand their complexity; location, functional capabilities, the antigens they recognize and how they are activated.

The development and tissue locations of different γδT cells are not directly comparable between humans and mice, and therefore care has to be taken when extrapolating from one to the other. In both cases, γδT cells are generated in the thymus from CD4^−^ CD8^−^ double negative (DN) progenitor cells, which commit to the αβ or γδT cell lineage depending on the type of V(D)J rearrangements and the strength of the pre-TCR signal (6, 7). In humans, the repertoire of Vδ and Vγ genes is much smaller than that for αβT cells (8), with Vδ1, Vδ2, and Vδ3 chains being the most frequently used Vδ gene segments. These can pair with one of the several functional Vγ gene segments; Vγ2, Vγ3, Vγ4, Vγ5, Vγ8, Vγ9, or Vγ11, although some combinations are more likely than others. In healthy human adults, the majority of γδT cells in peripheral blood are Vγ9Vδ2^+^ T cells, and typically represent between 1 and 10% of circulating lymphocytes. These cells can also be found as a minority in gut, liver and other epithelial tissues, whereas Vδ1^+^ γδ cells are present in higher frequencies at these sites (9).

In mice, DN progenitors in the thymus give rise to temporal waves of discrete populations of γδT cell precursors that populate distinct anatomical sites (6, 7, 10, 11). The first waves of γδT cells arise during embryonic development and bear invariant TCRs. Cells bearing the Vγ5Vδ1^+^ TCR or dendritic epithelial T cells (DETC) emigrate to populate the skin epidermis, and Vγ6Vδ1^+^ T cells will inhabit the reproductive tract, oral mucosa, peritoneal cavity and some other tissues, such as liver, lung, intestinal lamina propria, dermis etc. A third wave, produced at around birth, is characterized by Vγ7Vδ4^+^ TCRs, and populates the small intestinal epithelium. Subsequently, Vγ1^+^ and Vγ4^+^ γδT cells leave the thymus and recirculate between peripheral blood and lymphoid tissues, such as the spleen. These Vγ1^+^ and Vγ4^+^ γδT cells are the only γδT cells that are produced throughout life. Thus, for both species, the final tissue distribution of γδT cell subsets is related to a greater or lesser extent by their TCR chains (12).

The preferential location of different γδT cell subsets is important for understanding their role in malaria, where encounters with *Plasmodium* in the vertebrate host can occur in many different sites; skin, liver, peripheral blood and lymphoid organs. While γδT cell TCRs are distinct in human and mouse, it seems that in both cases γδT cells in tissue sites are different from circulating γδT cells, and some functions may be conserved across the two species [reviewed in (12)].

## γδT Cell Responses in Human and Mouse *plasmodium* Infections

**Figure 1:**
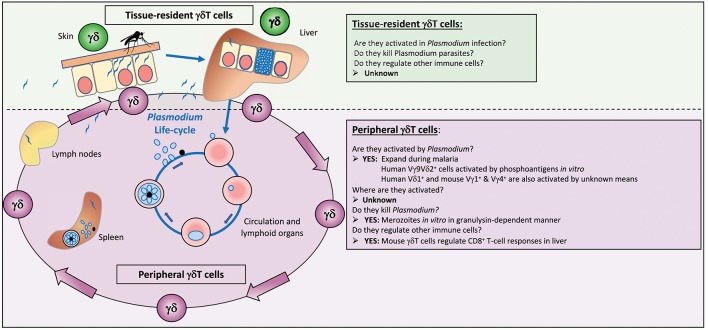
γδT cells in malaria. Infected mosquitoes inject *Plasmodium* parasites in the form of sporozoites into the skin of a susceptible host from where they migrate to the liver to find an appropriate hepatocyte for invasion and replication. Some of these sporozoites will end up in lymphoid organs, such as spleen and lymph nodes as well. The parasite in hepatocytes undergoes rapid multiplication to form merozoites, which burst out of the infected cell and enter the blood circulation where they infect red blood cells and initiate multiple rounds of maturation and replication until the immune system manages to eliminate the parasites from the blood. During all these different steps—passage from skin to liver to blood—γδT cells present in the tissues (both tissue-resident and circulating γδT cells) could recognize parasites and become activated. Circulating γδT cells become activated during malaria, but nothing is known about where they are activated, whether in the skin, the liver or the lymphoid organs, and whether they really contribute to the antiparasite response during a natural infection. Tissue-resident γδT cells in the skin and liver could become activated, and protect against a new infection. Even less is known about responses of tissue-resident γδT cell subsets, the antigens they recognize, and whether they are able to kill sporozoites or infected hepatocytes.

The malaria parasite is present in different locations during its life cycle in the vertebrate host: trafficking sporozoites in the skin, within hepatocytes in the liver, and a replicative cycle of invasion into, and egress from erythrocytes in peripheral blood with circulation through lymphoid organs, particularly the spleen (Figure 1). Encounters with γδT cells can therefore be multiple, and we need to incorporate our knowledge of different populations of these cells when considering their role in malaria: their recognition specificities, their locations, their possible effector or regulatory functions and their “memory” status, all of which could influence the outcome of a malaria infection (Table 1).

**Table 1:** γδT cells in malaria: human and mouse.

	**Human**	**Mouse**
**KNOWN** ***γ*δT CELL SUBSETS**^**a**^ **EXPANDED DURING** ***Plasmodium*** **INFECTION IN**		
Skin (sporozoite entry) Liver (liver-stage infection) Peripheral blood & lymphoid organs (blood-stage infection)	Not known Not known Vγ9Vδ2 (14–19), Vδ1 (20–24)	Not known γδT cells expanded (4); subset not known Vγ1 (13, 19, 25), Vγ2 (25–27), Vγ4 (25)
***γ*δT CELLS EXPANDED BY**		
Irradiated/Live sporozoites Liver-stage parasites Blood-stage parasites	Yes (1, 4, 28, 29) Not known Yes—*in vitro* by *P. falciparum* (14, 16, 30, 31)	Yes (4, 32, 33) Not known Yes—*in vivo*; variable dependent on *Plasmodium* spp. (13, 19, 26, 34, 35)
Antigen(s)	Vγ9Vδ2: possibly phosphoantigens (36) Vδ1: Not known	Not known
Co-stimulation requirements	CD28/CD80/86 (37), IL-2 (37), IL-15 (38)	CD28/CD80/86 (37), IL-2 (34, 37, 39–42)
**POTENTIAL EFFECTOR FUNCTIONS AGAINST**		
Sporozoites Liver-stages Blood-stages	Not known Not known Vδ1, Vγ9Vδ2: degranulation and granulysin decrease *P. falciparum* replication *in vitro* (38, 43, 44) IFNγ-production by Vγ9Vδ2 cells induced by *P. falciparum* iRBC *in vitro* (22, 28, 40, 43, 45)	Not known Not known IFNγ-production during blood-stage infection (27)
**PROTECTIVE AGAINST INFECTION BY**		
Sporozoites Blood-stages	Vγ9Vδ2 cell expansion associated with protection in irradiated sporozoite vaccination (1, 29) Not known	Yes - by recruitiment of CD8α+ dendritic cells which cross-present to effector CD8+ T cells (4) Lack of γδT cells: variable effect on parasitemia depending on *Plasmodium* spp. (19, 27, 35, 40–42, 46–48). Subset unknown Vγ1 γδT cells and M-CSF protect against chronic *P. chabaudi* (19)

a *γδT cell subset as determined by γ [mouse, Tonegawa 1986 nomenclature; (49)] or δ (human) T cell receptor chain expression*.

### γδT Cell Responses Against Sporozoites

Infection of humans with live sporozoites under chloroquine prophylaxis (28) or after immunization with irradiated sporozoites (1, 4) results in a long-lasting expansion of γδT cells with a “memory” phenotype. Although these cells are detected in peripheral blood, it is not known at which stage of the parasite life-cycle, or where, they were induced (skin, draining lymph nodes, infected hepatocytes or peripheral blood/spleen, see Figure 1 and Table 1), whether they recognize antigens expressed uniquely at the sporozoite stage, or even whether they were activated by parasite antigen *per se*. That they express Vγ9^+^Vδ2^+^ TCRs (1, 29) suggests that they may be circulating γδT cells with access to many tissues. In humans, their activation and effector site is difficult to establish. Although the subpopulations of mouse and human γδT cells are not directly equivalent, the similar preferential tissue locations, eg circulating Vγ4 and Vγ1 mouse γδT cells and Vγ9Vδ2^+^ human cells; tissue-resident Vδ1^+^ cells in humans and Vγ5^+^ and Vγ6^+^ in mice, are such that one could pursue this in mouse models.

Whether and how γδT cells interact with sporozoites in the skin is unknown, but given their appearance after sporozoite immunization, this would be an area of research worth pursuing. Rapidly activated γδT cells could either have some direct effector function or recruit other effector cells to prevent further development of the infection. The use of mice that lack epidermal DETCs or dermal Vγ6^+^ γδT cells (50, 51), may be useful tools to determine the importance of skin-residing γδT cells in malaria.

The association of γδT cells with protection after irradiated sporozoite immunization (1, 4) and the demonstration of the protective effects of γδT cells in irradiated sporozoite vaccination in *P. yoelii* and *P. berghei* mouse models (4, 32, 33) also suggest that these cells have an important protective role in the liver. The views are that γδT cells act either as effector cells that operate in the absence of αβT cells, or as accessory cells for appropriate protective responses from other cells (4). With the differences in the experimental approaches and the heterogeneity of γδT cell functions now known, it is likely that γδT cells could be performing both functions in these experimental models. It will also be important to determine whether intrahepatic (possibly Vγ6^+^) or blood Vγ1^+^ or Vγ4^+^ γδT cells are playing the protective role (52). In Zaidi et al.'s mouse model, the Vγ4^+^ γδT cells do not appear to have a role in protection, while the nearest equivalent in humans, blood Vγ9Vδ2^+^ cells are found to associate with protection. We have some clues about γδT cells induced by pre-erythrocytic stages of *Plasmodium* but many questions remain: which γδT cells? Where are the cells activated? What do they recognize? If they are accessory cells how are they functioning? If they are direct effector cells, what is their mechanism? The mouse models will be a good way to address these issues.

### γδT Cell Responses Against Blood-Stages

The first observations of γδT cell responses to blood-stage malaria parasites were made more than 25 years ago, and showed that Vγ9Vδ2^+^ γδT cells of malaria-naïve individuals proliferate in response to *P. falciparum*-infected red blood cells (iRBCs) *in vitro* (14, 16, 30, 31). Following this, it was demonstrated that Vγ9Vδ2^+^ cells increase to up to 10–30% of total PBMC in *P. falciparum-* or *P. vivax-*infected adults with little or no previous exposure to malaria (14–18). More recently, this has also been shown in experimentally infected individuals (19). In regions of high malaria endemicity, or after multiple infections, healthy individuals already have a higher frequency of γδT cells than European populations (53), and there is no further peripheral expansion of Vγ9Vδ2^+^ γδT cells on exposure to the parasite; however, there is a large increase in the proportion of Vδ1^+^ cells in the PBMCs (20–24). The reasons for the relative expansion of Vδ1^+^ cells is not understood but could be due to the retention of active Vδ2^+^ cells in the spleen, thus changing the proportions in peripheral blood, or to the circulation of Vδ1^+^ cells normally activated and residing in tissues, such as liver and skin.

γδT cells also increase in most of the rodent models of malaria studied. They are expanded in spleens of mice infected with different strains of *P. chabaudi, P. yoelii, and P. berghei* within 1–2 weeks of a blood-stage infection, depending on the mouse/parasite combination, reaching a peak at 3 weeks post-infection in non-lethal infections (13, 19, 34, 35), but with no expansion in a lethal *P. yoelii* infection (26).

The advantage of mouse models is that they can tell us whether the γδT cell response observed during blood-stage infection plays any protective role. In all the different infections studied—*P. yoelii* XNL,XL, *P. berghei* XAT, *P. chabaudi* AS, AJ, *P. chabaudi adami* K556A—mice without functioning γδT cells due to *in vivo* depletion with specific antibodies, or because of targeted deletion of the δ gene, show exacerbated acute parasitemias (although the increase is more pronounced with *P. yoelli* than with, eg, *P. chabaudi*) (19, 26, 27, 40, 41, 46–48), and in some cases this results in a lethal infection (26, 42, 48). In *P. chabaudi*, depletion of γδT cells additionally results in delayed clearance (27, 40, 41, 46, 47), or an increased magnitude of chronic parasitemias (19).

It is important to know which subpopulations of γδT cells in the mouse are responsible for the protective effect, as this could give us clues about where the γδT cells may have been activated as well as the nature of the inducing antigens. The γδT cell response to blood-stage *Plasmodium* in humans is dominated by cells bearing Vγ9Vδ2^+^ TCR-chains, and thus one might predict that the nearest mouse counterparts are the blood/lymphoid circulating Vγ1^+^ or Vγ4^+^ γδT cells. However, the mouse response, at first glance, appears to be more heterogeneous. Although most reports show the circulating γδT cell bearing Vγ1^+^, and/or Vγ4^+^ TCRs to be expanded with different δ chains (13, 19, 25), there are also reports of Vγ2^+^ T cells (25, 27, 35). This could reflect the very different infections of *P. chabaudi, P. yoelii* and *P. berghei* strains in the mouse, and the different mouse strains used. However, some of the differences between different studies may be due to the confusing systems of nomenclature used for designating the γ chain of the mouse γδTCR (49, 54–56). Many of the earlier papers do not define clearly the nomenclature used. Given the contribution of mouse γδT cells to the control of blood-stage infections in mouse models, it would be well worth revisiting this, and reanalysing the γδT cell response in the different blood-stage infections.

All the blood-stage mouse infections described here differ in one major respect from natural infection, or sporozoite vaccination, in that they are not initiated via the bite of an infected mosquito. Not only does this mean that two key sites of potential γδT cell activation, skin and liver, are missing, but also the parasites from serial blood passage may differ in their transcriptional profile and in virulence (57, 58). The lack of the pre-erythrocytic stages of *Plasmodium* could influence location, specificity and dynamics of γδT cell activation. While the mouse γδT cell subsets are not direct counterparts of human, if we are to use the mouse model to elucidate mechanisms of γδT cell activation and protection, we should approximate as closely as possible to the mode of infection in humans.

## γδT Cells: Antigen Recognition and Activation

Unlike αβT cells, antigen recognition by the γδTCR is not restricted to the classical major histocompatibility complex (MHC). Some γδT cell subsets do recognize members of the MHC superfamily or MHC-like molecules, such as CD1, other γδT cell subsets recognize full proteins or unique pathogen-associated molecular patterns (both of foreign and self-origin), whereas for other γδT cells, probably the vast majority of them, we do not have any idea of the type of antigen they recognize (5). The lack of diversity amongst V chain composition, especially in tissue-resident γδT cells, suggests that foreign antigen may not be the primary target of these cells, and suggests a role for γδT cells in lymphoid stress surveillance, perhaps with self-stress molecules representing the primary γδTCR-ligands (5). Which begs the question what do they recognize in a *Plasmodium* infection?

Human peripheral blood Vγ9Vδ2^+^ γδT cells recognize phosphoantigens, the most potent being (E)-4-hydroxy-3-methyl-but-2-enyl pyrophosphate (HMBPP), an intermediate in the alternative non-mevalonate pathway of isoprenoid biosynthesis. HMBPP is essential for the production of sterol-containing biomolecules including cholesterol, heme and steroid hormones (5, 8). This pathway is used by *Plasmodium* spp. and other apicomplexa, as well as plants and bacteria, but not by higher eukaryotes, suggesting that these parasite products could stimulate Vγ9Vδ2^+^ T cells without compromising self-tolerance. A soluble molecule with the same characteristics is produced by mature blood-stage *P. falciparum* (http://plasmodb.org; Gene ID: PF10_0221), and secreted during parasite egress (36). However, it has not been directly shown that HMBPP is responsible for Vγ9Vδ2^+^ T cell activation in *Plasmodium* infection. Isopentenyl pyrophosphate (59), which can be produced by higher eukaryotes in the mevalonate pathway, is similar to but less potent than HMBPP in activating Vγ9Vδ2^+^ T cells (5, 8), and could be another source of phosphoantigens during *Plasmodium* infection.

How Vγ9Vδ2^+^ cells interact with phosphoantigens is not clear, as no direct contact with soluble/secreted HMBPP has been described. As the cells need cell contact for their activation, it is likely that an additional cell-surface molecule on the target is needed. Butyrophilins eg BTN3A or CD277, play an important role in the activation of Vγ9Vδ2^+^ cells in response to phosphoantigens. One proposal is that BTN3A works as a cell surface antigen-presenting molecule. More recently it has also been proposed to work as an intracellular detector of phospoantigens that is capable of translocating to the cell surface to stimulate Vγ9Vδ2^+^ T cells (60, 61). These findings suggest that Vγ9Vδ2^+^ T cells may not need direct interaction with iRBCs for their activation. As we do not know where they recognize their specific antigens, and where they could carry out any effector functions, we can only speculate on the source of antigen in malaria.

There is no direct counterpart for Vγ9Vδ2^+^ cells in the mouse, and there is no evidence that the circulating γδT cells of the mouse recognize phosphoantigens, therefore it is difficult to compare TCR specificities. Perhaps the best use of the mouse models would be to discover where these circulating γδT cells are activated and carry out their functional activities, rather than in a search for antigen specificity.

Human γδT cells expressing the Vδ1 chain and different γ chains, are abundantly present in tissues and normally form a minority in peripheral blood. Nevertheless, their frequency is increased in *Plasmodium* infections in areas where malaria is hyperendemic (20–24), and in other infections, such as HIV (62) and in the liver in HCV (59). The TCR of Vδ1^+^ γδT cells has an oligoclonal repertoire distinct from that of circulating γδT cells (5, 63). These cells are highly enriched in epithelial tissues, can recognize a range of epithelial tumors, possibly through recognition of stress-induced MHC class I-related molecules MICA and MICB, can respond to autologous and/or endogenous phospholipids presented by CD1, and display TCR-driven clonal expansions in response to Cytomegalovirus (along with the minor Vδ3^+^ and Vδ5^+^ subsets), and possibly HIV as well as malaria (64, 65). This raises questions about why and how the Vδ1^+^ T cell subset is expanded in Africans (20–22, 53). There is no evidence that they respond to *Plasmodium*-iRBCs *in vitro* (14), but of course there may be other activation requirements not provided in tissue culture, e.g., interleukin-2 (IL-2). We can only guess at the precise ligands that could activate these “epithelial” γδT cells in malaria. A strong possibility could be that they are recognizing stress-related molecules perhaps in the liver or skin as a result of pre-erythrocytic infection, or in the liver as a result of coping with chronic blood-stage infections. It is currently not known whether mouse DETCs and Vγ6Vδ1^+^ are activated and expand during primary or repeated *Plasmodium* infections, or which antigens they would recognize in such a situation.

In addition to their TCR, γδT cells express other receptors as well, including toll-like receptors, CD16, CD226, natural killer receptors, and NKG2D (24, 66–68). Whether and which co-receptors are engaged in response to *Plasmodium* is an open question, and worthy of investigation as it may help explain how/why different γδT cells become activated in malaria.

γδT cells are rapidly activated and do not necessarily require a lymphoid environment. However, they do need costimulation for their proliferation and survival (37, 69). For human and mouse γδT cells responding to *Plasmodium*, costimulation is provided via interaction of CD28 on the γδT cell and CD80 and CD86 on the target/presenting cell. IL-2 appears to be a requirement for γδT cell activation to *Plasmodium*, either through an autocrine loop via TCR signaling (37, 41), or through the exogenous IL-2 provided by CD4^+^ T cells (34, 39, 40, 42) and IL-15 can augment the IL-2-dependent response (38). A deeper understanding of the requirements for activation and maintenance of different γδT cells in malaria would be important for determining how to harness these cells for protective immunity.

## Functional Responses of γδT Cells in Malaria

It is becoming clear that γδT cells are more complex and varied in their immune roles than originally thought. Much of the work on function and roles of γδT cells has been carried out in mouse models, and although some aspects of γδT cells clearly vary between species, critical roles in early immune responses are often conserved. Common features of γδT cells include innate receptor expression, antigen presentation, cytotoxicity, and cytokine production (Table 1). However, the functional plasticity of Vγ9Vδ2^+^ cells of humans observed *in vitro* or *ex vivo* (9) is not seen in mouse γδT cells, where cytokine profile is predetermined in the thymus and by their final tissue location (7, 9, 33, 70, 71).

### Cytokine Production and Cytotoxicity

In humans and mice, rapidly activated blood/circulating/lymphoid γδT cells produce large amounts of interferon-γ (IFN-γ) after stimulation *in vitro* with *P. falciparum*-iRBCs (22, 28, 31, 43, 45) or during early blood-stage infection with *Plasmodium* (27). IFN-γ can mediate killing of parasites in infected liver cells (72), and more indirectly, activate phagocytes that can eliminate blood-stage parasites by antibody-dependent or -independent mechanisms (73). In the absence of αβT cells, γδT cells are required in some irradiated sporozoite immunization protocols to eliminate liver-stage parasites in mouse models (28, 32, 33, 45). However, it is not clear whether IFN-γ from γδT cells is crucial for these effector mechanisms in malaria, as αβT cells and NK cells also produce this cytokine. On the other hand, loss of γδT cells in most mouse models of malaria does not compromise greatly the ability to remove acute blood-stage infections, although they may be important to control recrudescences (19, 26, 27, 32, 40, 41, 46–48).

Through their cytotoxic activity, it is conceivable that γδT cells can directly kill parasites (5). Both activated human Vδ2^+^ and Vδ1^+^ T cells degranulate when incubated with free merozoites, but not intraerythrocytic parasite stages, and can inhibit *P. falciparum* replication *in vitro* in a dose-dependent manner (43, 44, 74). This direct parasiticidal effect is dependent on granulysin rather than perforin, and requires contact or at least close proximity to target cells. Furthermore, patients infected with *P. falciparum* had elevated granulysin plasma levels and high numbers of granulysin-expressing Vγ9Vδ2^+^ T cells, which degranulated when incubated in the presence of iRBCs (44). How effective these cytotoxic responses are *in vivo* remains debatable since merozoites only spend a very short time in the extracellular environment between egress and re-invasion. It might be more effective in tissues with a low blood flow, such as the red pulp of the spleen, where both γδT cells and mature iRBCs are highly prevalent and thus would have a higher chance of an encounter (75). Whether γδT cells can directly kill merozoites in mice, and whether this contributes to control of parasites has not yet been demonstrated. Perhaps the use of conditional knock-out mice in which the cytolytic machinery or IFN-γ-signaling has been specifically ablated in γδT cells or CD3DH mice which have reduced numbers of IFN-γ-producing γδT cells (71) would elucidate their roles more clearly.

There is now a wealth of literature about the involvement of different subpopulations of γδT cells in MHC class I presentation, regulation of other immune cells, and production of cytokines important for myeloid cell development. The most widely studied human γδT cells, Vγ9Vδ2^+^ cells, have a wide variety of other functions including follicular helper-like, Th17-like, and Th2-like responses (9). Most of these aspects have not been explored in detail in malaria, but mouse models could offer good insights into how they contribute to the protective host response to *Plasmodium*. A recent example of an immunoregulatory function of γδT cells is the study by Zaidi et al. (4), where they have shown that γδT cells are important for the protective response induced by irradiated sporozoites, but it seems not as direct effectors. They are required for the development of an effective CD8^+^ T cell response. In this immunization model, γδT cells were required for recruitment of cross-presenting CD8α^+^ dendritic cells, necessary to activate effector CD8^+^ T cells. How this is achieved is currently not known, but a recent paper on the interplay of γδT cells and myeloid cells may offer some clues. γδT cells producing macrophage-colony stimulating factor are important for controlling the chronic phase of *P. chabaudi* infections, suggesting that γδT cells are interacting with the myeloid cell compartment to control parasitemia (19, 76).

The functional capacities of epithelial γδT cells have not been investigated in great detail in malaria. As mouse skin and liver γδT cells produce IFN-γ and/or IL-17, and human skin Vδ1^+^ cells, in addition to production of IFN-γ, can be cytotoxic, and these cells, when activated, recruit myeloid cells, and enhance phagocytosis (77, 78), such studies would be worthwhile.

### γδT Cell “Memory” Responses

Long-term responses, or effective reactivation on second encounter with antigen requires some form of longevity of the cell population, either by constant re-stimulation, or through development of long-lived memory cells. Obviously for harnessing γδT cells in protective immune responses induced by vaccination it would be good to have an expanded population of “memory” cells that give an enhanced and more rapid response. The general view has been that γδT cells, although expressing TCRs encoded by somatically rearranged genes, are innate-like effectors that do not establish antigen-specific memory. It could also be argued that there is no need for the development of memory cells, as γδT cells have a relatively limited repertoire of TCRs which respond rapidly to the same set of antigens without the need for massive expansion, and this would happen on every exposure to appropriate antigens (79). Nevertheless, there are reports of adaptive-type memory responses of γδT cells. Human Vγ9Vδ2^+^ T cell responses to phosphoantigens are increased by prior *Mycobacterium bovis* BCG vaccination (80). *In vivo*, there is a long term expansion of effector memory Vδ2^−^ cells in human Cytomegalovirus infections (81) and enhanced “secondary” responses by Vγ9Vδ2^+^ T cells in macaques infected with live Mycobacteria (82). Mouse “memory-like” Vγ6^+^ γδT cells were maintained for more than 5 months in mesenteric lymph nodes after *Listeria monocytogenes* infection (83) and Vγ4^+^ γδT cells have been found to persist in dermis and draining nodes for more than 3 months in a skin inflammation model (56, 84). Long-term elevation of γδT cells has been observed in peripheral blood of *P. falciparum*-exposed humans under chloroquine prophylaxis (28) or following irradiated sporozoite vaccination (1, 4), and in humans in malaria-endemic areas (53). Whether these contain true long-lived memory cells able to exist in the absence of antigens is not known. Such studies have not yet been carried out in either human or mouse *Plasmodium* infections.

## Summary

We have tantalizing evidence that γδT cells are important in the protective immune response to *Plasmodium*, particularly those induced by whole organism vaccination. It is also clear that we know little about which γδT cells are important, where and how they are activated and exactly how they contribute to protective immunity. Studies investigating γδT cells in the skin and liver, and especially mechanistic studies on the function of γδT cells in malaria are scarce or even lacking. Mouse models can help with several of these aspects, and now is the time to invest in this important part of the host response to *Plasmodium*.

## Author Contributions

KD and JL designed and drafted the manuscript.

### Conflict of Interest Statement

The authors declare that the research was conducted in the absence of any commercial or financial relationships that could be construed as a potential conflict of interest.
